# Overview of Canada’s Answer to the COVID-19 Pandemic’s First Wave (January–April 2020)

**DOI:** 10.3390/ijerph18137131

**Published:** 2021-07-03

**Authors:** Deborah Urrutia, Elisa Manetti, Megan Williamson, Emeline Lequy

**Affiliations:** 1 Institute of Global Health, University of Geneva, 1202 Geneva, Switzerland; deborah.urrutia@etu.unige.ch (D.U.); megan.williamson@etu.unige.ch (M.W.); 2 Centre de Recherche du Centre Hospitalier de L’université de Montréal, Montréal, QC H2X 0A9, Canada; e.lequy@gmail.com

**Keywords:** COVID-19, Canada, health system, interventions, capacity building, impact

## Abstract

Canada is a federal state of almost 38 million inhabitants distributed over ten provinces and three territories, each with their own power regarding health. This case study describes the health infrastructures’ situation before the COVID-19 outbreak and their adaptations to face the expected cases, the available epidemiologic data for the beginning of the first wave (January–April 2020), and the public health and economic measures taken to control the pandemic both at the federal level and breaking down by province and territory. Canadian health infrastructures offered on average 12.9 intensive care units beds per 100,000 (occupancy rate ~90% before the outbreak), unevenly distributed across provinces and territories. Canada implemented public health measures, such as social distancing, when hospitalization and death rates due to the pandemic were still lower than in other countries; each province and territory adapted and implemented specific measures. Cumulated cases and deaths substantially increased from mid-March 2020, reaching 65 cases and 2 deaths per 100,000 on April 12, with strong differences across provinces and territories. Canada has been affected by the COVID-19 pandemic’s first wave with a generally slower dynamic than in the USA or in the European Union at the same period. This suggests that implementation of public health measures when health indicators were still low may have been efficient in Canada; yet the long-term care sector faced many challenges in some provinces, which drove a large part of the pandemic indicators.

## 1. Introduction

In December 2019, a new infectious disease was identified in Wuhan (China), caused by a coronavirus named SARS-CoV-2 (short for Severe Acute Respiratory Syndrome Coronavirus 2), and the World Health Organization named this quickly spreading disease COVID-19. At this time, SARS-Cov-2 had just been identified, but other coronaviruses such as SARS-CoV or MERS-CoV (short for Middle-East Respiratory Syndrome Coronavirus) had already caused severe respiratory illnesses in outbreaks in 2002 and 2012, respectively. The most common symptoms of COVID-19 include fever, shortness of breath, dry cough, and fatigue. Most people will develop moderate respiratory symptoms, which will not require any specific treatment; some people will even be asymptomatic [1]. However, SARS-CoV-2 can induce very severe consequences on the respiratory system, with difficulties to breathe to the point of acute respiratory distress syndrome and the need for hospitalization [2].

Because of the novelty of this virus, research quickly organized to find causes and solutions to the spread of the virus, as well as possible treatments and vaccines. In the first wave, the virus was thought to be mainly transmitted through small droplets from the nose or mouth of an infected person or from direct contact (“fomites”)—though later research acknowledged transmission also occurred through aerosols [3,4]—a transmission occurring mainly in indoor, crowded and insufficiently ventilated spaces [2].

COVID-19 was declared a pandemic by the WHO on 11 March 2020. Yet, not all countries have been affected the same way at the same time. About one month after the first infected patient was declared in China, the virus had reached North America. However, then, the trajectories of cases, hospitalization and death counts in Canada differed from those in many occidental countries. Besides, Canada has specificities as a federal state making it interesting to focus on in this case study, which aims to understand how Canada dealt with the COVID-19 pandemic given the unexpected circumstances and rapid spread of the coronavirus.

After briefly presenting Canada according to its geographic, demographic, economic and political characteristics, we will describe Canada’s health system (including the long-term care sector) and its adaptation to the COVID-19, the available epidemiologic data, and the series of public health measures taken throughout the country in response to the COVID-19 at the beginning of the first wave (January–April 2020).

## 2. Case Presentation

### 2.1. Demographic, Economic, Geographic, Political and Climatic Characteristics of Canada

**Figure 1:**
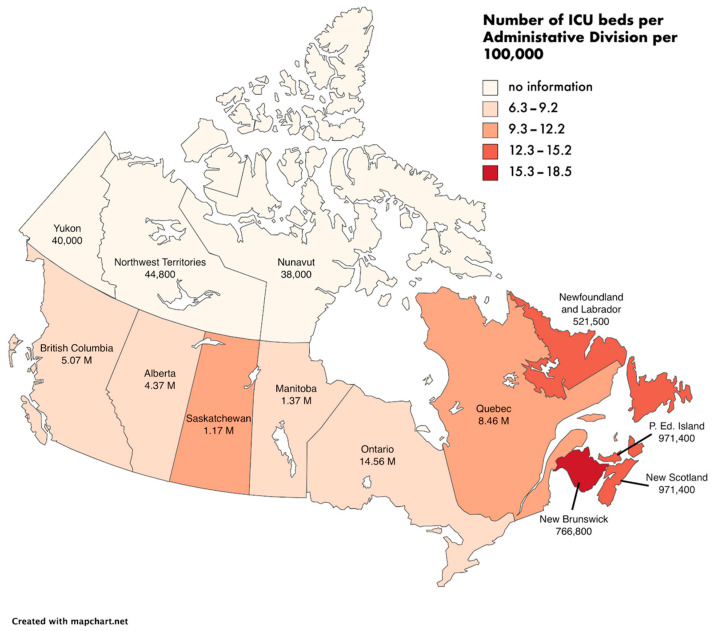
Map of the intensive care units (ICU) beds available per 100,000 by Canadian administrative division. Below the name of each administrative division is the number of inhabitants (M stands for millions).

Canada is a federal state with the second largest area in the world (almost 10 million km^2^); in 2020, its population was about 38 million, unequally distributed across its ten provinces and three territories, which will be altogether referred to as administrative divisions in the rest of this case study (Figure 1).

More than 85% Canadians live in four provinces: Ontario (38.6%), Quebec (22.6%), British Columbia (13.5%), and Alberta (11.6%) [5], and mostly along the southern border of Canada characterized by milder climatic conditions than the northerner areas characterized by extreme cold and long winters, and therefore less inhabited. Canada also includes about 5% of indigenous peoples (First Nations, Metis, and Inuits) unequally distributed across administrative divisions, from less than 3% of the population in Ontario or Quebec, to between 20 and 80% of the population in the three territories. Some parts of Canada are on boil-water advisory or cannot always access drinking water, and some are very remote and isolated—especially the indigenous people. Such poor local access to clean water and subsequent poor access to hygiene and handwashing was considered very important in the first wave especially because SARS-CoV-2 was thought to transmit through “fomites”, and some faecal-oral transmissions may occur [6,7].

These characteristics have consequences on the health system’s operations (see Section 2.2) and responses to the COVID-19 pandemic (see Section 2.3).

The economic situation of the country also differs across administrative divisions, according to their own history of migration, economic development, industrial location, urbanization and land use—including the access to waterways and natural resources such as forests and petroleum.

The political system of Canada is a constitutional monarchy and a parliamentary democracy, with three levels of government, each with their own responsibilities: (i) the federal government or the government of Canada, (ii) the provincial and territorial governments, and (iii) the municipal or local governments. The federal government includes the Queen Elizabeth II represented by the governor general, the executive branch (including Canada’s Prime Minister), and the Parliament. Each province or territory has key powers, such as the provision of fundamental social services (including health), and power over local government [8].

### 2.2. Health Care System

#### 2.2.1. Generalities

The Canadian health system provides access to publicly funded health services to its entire population. Universal coverage is a strong principle and an objective of this public health policy. According to one of the main principles of the Canada Health Act (the federal legislation for publicly funded health care insurance), each person living on the country’s territory is provided with public care insurance that covers all medical treatment considered necessary. The Canada Health Act establishes the criteria and conditions related to the health services that must be provided by the provinces and territories. The Federal Government’s role is to set these principles nationally. It also delivers services for the most remote or vulnerable people, such as First Nations people living on reserves, Inuits, refugees, etc.

Then, it is mostly the provincial or territorial government’s responsibility to deliver the adequate healthcare to its population. Thus, the level of services may vary across administrative divisions, which show differences in the quality of healthcare even in similar socio-economic environments [9]. For these reasons, in the following sections, we will describe the health system capacity, the COVID-19 situation, and the measures taken to deal with the pandemic both at national level and at provincial or territorial level.

Canada’s health expenditures amounted to more than CAD 265 billion in 2019, or CAD 7064 per Canadian, therefore representing 11.5% of Canada’s gross domestic product that year [10].

#### 2.2.2. Health Infrastructure Capacity before COVID-19

In 2020, Statistics Canada reported 1140 hospitals over the country, with between 3 and 5 in the territories and between 9 (Prince Edward Island) and 349 in Quebec [11]. In 2019, in terms of healthcare workers, Canada included 124.5 family medicine physicians per 100,000 (unevenly distributed across administrative divisions from 44.3 per 100,000 in Nunavut to 165.5 per 100,000 in Yukon), 33.2 respiratory specialists per 100,000 (from 7.8 per 100,000 in Nunavut to 55.7 per 100,000 in New Brunswick), and 118.0 pharmacists per 100,000 (from 98.8 per 100,000 in the Northwest Territories to 163.1 per 100,000 in Yukon) [12]. Finally, there were 2039 long-term care homes of which 46% were publicly owned and 54% were privately owned (less than a half of them were not-for-profit), among which 17 in the territories (all publicly owned), and, in each province, from 36 (Newfoundland and Labrador) to 626 (Ontario) (see Section 2.3.2 for a focus on COVID-19 in these facilities) [13].

As an indicator of the health facilities’ capacity to admit new patients with severe symptoms, we chose the number of intensive care unit (ICU) beds available. According to data from the Canadian Institute for Health Information, Canada had on average 12.9 adult ICU beds per 100,000 population in 2013–2014, a number within the range of various occidental countries such as France, Belgium, and Spain [14]. A total of 3170 ICU beds were estimated capable of invasive ventilation across Canada [15]. Before the outbreak of COVID-19, the hospital bed occupancy was around 90% [14].

Since the health infrastructure capacity and access varies across Canada’s administrative divisions, it is important to break down the national average (Figure 1): in 2019, ICU beds ranged between 6.3 (in British Columbia) to 18.5 (in Newfoundland and Labrador) per 100,000 population (without considering the three territories for which we found no official number—see below), with rural-urban disparities [10,11,15,16]. However, these ICU beds number represent the minimal health infrastructure capacity, since in an emergency situation like the COVID-19, bed restructuration enables health facilities to provide more beds in the intensive care units (see Section 2.2.3); however, the current availability of ICU beds still gives a good indication of the capacity of Canada to deal with an increasing number of new patients at the provincial level.

The three territories (Northwest Territories, Nunavut, and Yukon) seemingly had the least number of ICU beds with 5.5 per 100,000 of all Canada [17], and are also the administrative divisions with the smallest population and population densities and with the most difficult access; thus, Nunavut, the biggest administrative division of Canada with more than 2 million km^2^, has an average population density of only 0.02 people/km^2^. Even though most of the population is living in the main capital city, not all people can easily access hospitals all year round.

On the other side, provinces such as Ontario or Quebec have the largest population, but the number of available ICU beds per person is not the highest of Canada, with 7.9 and 10.5 per 100,000 inhabitants, respectively [15].

#### 2.2.3. Adapting the Health System to COVID-19

The Public Health Agency of Canada issued a first interim guidance for the clinical management of patients with COVID-19 in early April 2020 [18]. To prevent health infrastructures from being overwhelmed with new infected cases, Canadians have been asked to avoid social contact since mid-March 2020 (see Section 2.4), as it had been defined by scientists across the world as an efficient way to stop the spread of coronavirus. The objective was to slow down the number of infected people to give medical infrastructures time to admit new patients in need without running out of space and equipment—also known as “flattening the curve”.

Medical infrastructures sought to increase their capacity as follows.

First, they reset their priorities regarding the availability of beds and healthcare professionals and they made sure that health care professionals could staff all available beds and ventilators. Thus, across the country, nurses and physicians from other departments were trained to work in intensive care units, in case of need. For example, a collaboration of clinicians, educators, and scientists from the University of Toronto and the Toronto Academic Health Sciences Network developed an educational website with a training guide called “quick ICU training for COVID-19”. This guide provides training resources to prepare health professionals who are not accustomed to working in intensive care units but might be required to do so during the pandemic [19].

To optimize the hospital capacity across the country, tens of thousands scheduled surgeries have been cancelled or postponed [20]. On March 16, British Columbia Ministry of Health took the decision “to postpone non-urgent scheduled surgeries to ensure capacity in [their] hospitals to address COVID-19 patient needs” [21], which applied for more than 30,000 nonurgent operations between March 16 and May 18.

To avoid overwhelming hospital’s emergency departments, off-site screening facilities were set up, such as temporary testing clinics in Ontario. At the end of the study period (mid-April 2020), and globally in Canada, visits to emergency departments declined by a half [10].

Some alternatives were implemented to allow people to be taken care of while staying at home. For instance, regarding COVID-19, on March 21 the Canadian government launched an online self-assessment tool allowing people to detect their own symptoms and helping them decide whether they should seek for medical attention and when [22]; drive-through centers were set up so people could be tested for coronavirus without getting out of the car. Regarding health in general, doctors also used telehealth and virtual medicine to remotely assess their patients.

Finally, from mid-April, the federal government started to encourage the development of faster and more reliable tests, supported by the academics and industrial research experts [23].

It is worth mentioning that in 2003, Canada experienced the SARS epidemic caused by the above-mentioned SARS-CoV. This SARS epidemic resulted in thousands of Canadians in quarantine and the death of 44 people, thereby affecting Canada more than many countries outside Asia [24]. This first SARS outbreak had highlighted several weak points of the healthcare system against epidemics, and the lessons learnt from this epidemic (as described in the report “Learning from SARS”) prepared Canada to face COVID-19, at least in the sectors affected by SARS in which improvement were made—around 80% of the report’s recommendations were addressed since 2003 according to expert David Naylor [25,26,27]. Yet, some challenges remained and affected the dynamics of the COVID-19 in Canada as described below.

### 2.3. Epidemiological Situation of the Country Regarding COVID-19′s First Wave

#### 2.3.1. Epidemiological Summary (Dates)

**Figure 2:**
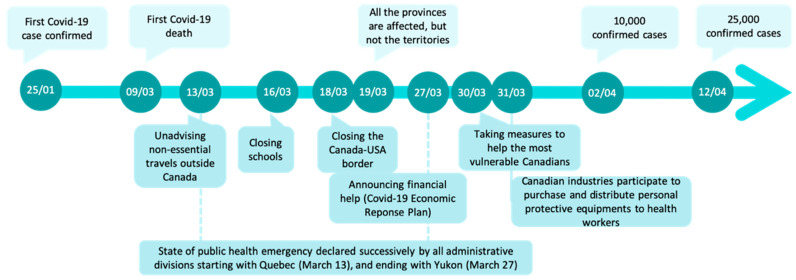
Timeline summarizing some key epidemiologic points (above the arrow) and public health or economic measures (below the arrow) in the study period.

Canada confirmed its first COVID-19 case in Toronto (Ontario) on January 25 (Figure 2), due to a trip to Wuhan, China. Afterwards, the COVID-19 affected the country differently depending on the administrative divisions as described below.

**Figure 3:**
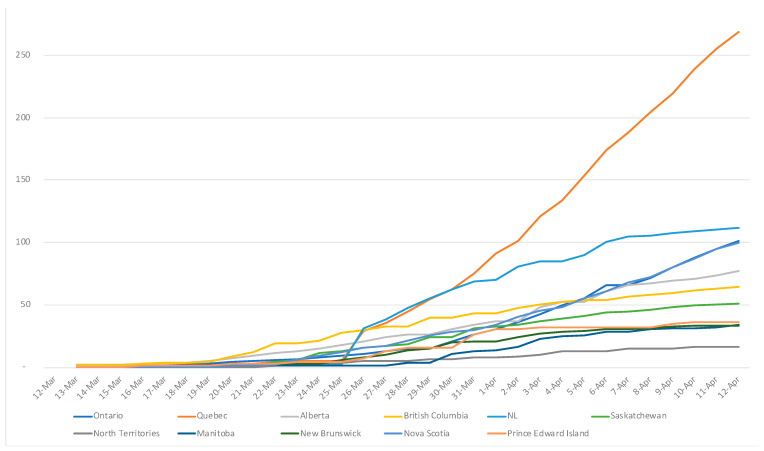
Positive cases per 100,000 by administrative division. The three territories (Nunavut, Yukon, and Northwest Territories) are regrouped together under “North Territories”, and NL stands for Newfoundland and Labrador.

The most likely exposure of reported cases changed over time, from being mainly due to traveling or being in contact with a traveler, to mainly due to community setting—this shift was reported to have occurred on March 24. Regarding traveling or being in contact with a traveler, the most important exposure risks likely occurred in airports. Canada’s main airports are located in Ontario (Toronto Pearson International Airport) and British Columbia (Vancouver International Airport), which were indeed the first provinces affected by the virus in February (Figure 3).

**Figure 4:**
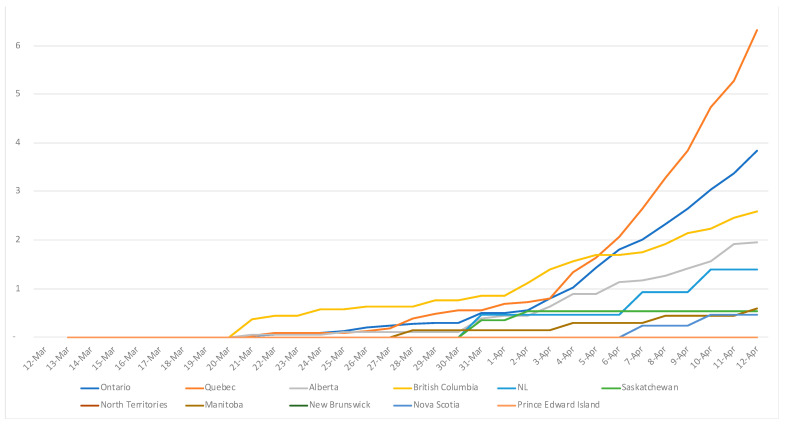
COVID-19 deaths per 100,000 by administrative division. The three territories (Nunavut, Yukon, and Northwest Territories) are regrouped together under “North Territories”, and NL stands for Newfoundland and Labrador.

The most affected province was Quebec with the steepest exponential curve and the most important number of confirmed cases (Quebec was also the province conducting the highest number of tests). The less populated territories, Yukon and Northwest Territories, were the least affected with 6 confirmed cases and 0 death at the end of March (Figure 4).

**Figure 5:**
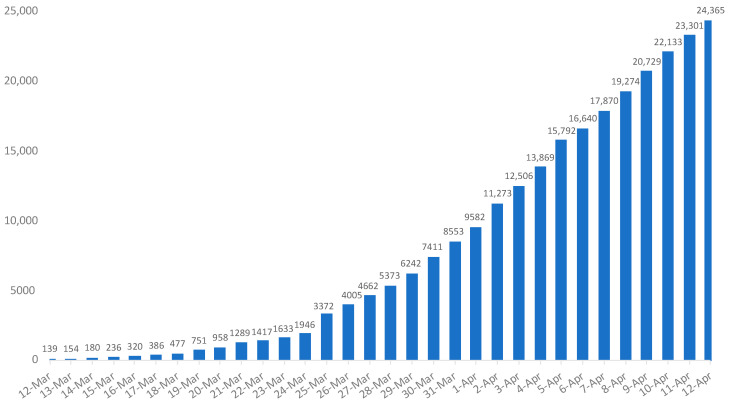
Daily cumulative positive cases of COVID-19 in Canada from 12 March to 12 April 2020.

On April 12, the country had confirmed almost 25,000 cases of COVID-19 with a rapid increase since the end of March (Figure 5).

**Figure 6:**
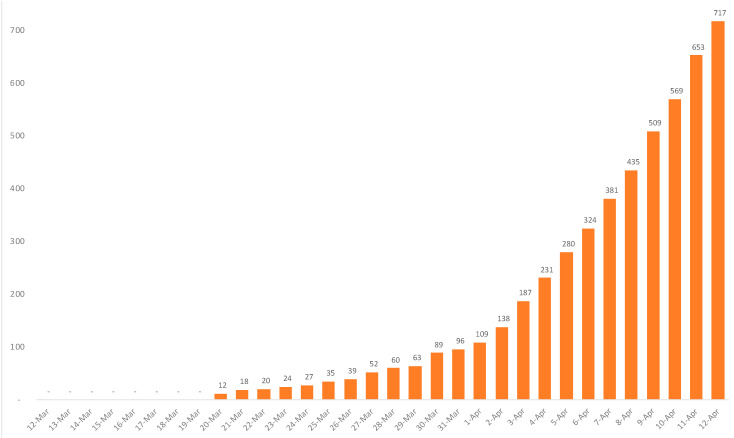
Daily cumulative COVID-19 deaths in Canada from 12 March to 12 April 2020.

The four most populated provinces of the country accounted for 93% of all confirmed cases in Canada, with ca. 52% in Quebec, 28% in Ontario, 6% in British Columbia and 7% in Alberta. The cumulative number of deaths exponentially increased up to 717 in April 12 (Figure 6); only 2% occurred outside these four most populated provinces [28].

**Figure 7:**
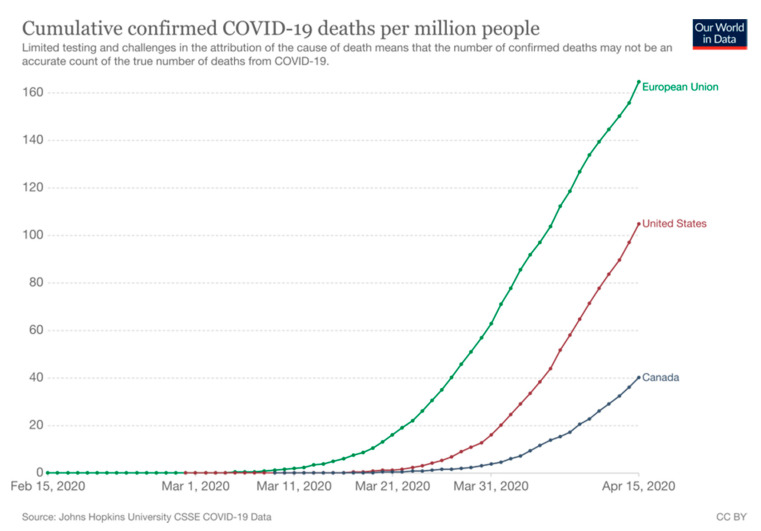
Cumulated confirmed COVID-19 deaths per 1,000,000 in Canada, in the European Union and in the USA between February and April 2020 (data from Our world In Data [29,30]). Raw data on confirmed cases and deaths for all countries is sourced from the COVID-19 Data Repository by the Center for Systems Science and Engineering (CSSE) at Johns Hopkins University.

Not all administrative divisions had the same capacity to test their population, and this must be considered when retrospectively analyzing the reported numbers [9]. Compared to other countries, the mortality rate was lower in Canada than in the European Union or the USA [29,30] (Figure 7).

#### 2.3.2. Focus on the Long-Term Care Sector

As of April 2020, ca. 46% of deaths related to COVID-19 occurred in long-term care homes in some provinces [31]. In April this rate was almost similar in Quebec (80%) and Ontario (70%). In June, it reached 81%, a substantially higher number than that observed in comparable countries (66% in Spain, 31% in the United States and 28% in Australia) [32].

Studies and report on this issue pointed out the outdated state of some of the infrastructures, the lack of information that would have allowed for good coordination of actions, and the crisis in the workforce in this sector. In addition, both public and private long-term care facilities had fewer staff, and staff lacked personal protective equipment (e.g., medical masks). Long-term care sector had not been affected by the above-mentioned SARS epidemic and not as many lessons could have been learned in this sector as in other sectors [25,27,33].

#### 2.3.3. Focus on Visible Minorities

In line with the general lack of race-based data in the health sector Canada, data on COVID-19 was not systematically broken down by race. Therefore, to date, there is no large-scale study on the consequences of COVID-19 by race or ethnicity. Yet, Statistics Canada was able to indicate that “COVID-19 mortality rates were higher in Canadian neighbourhoods with higher proportions of population groups designated as visible minorities” [34]. To refine this analysis, Statistics Canada focused on such neighbourhoods with Black population in Montreal (Québec), and with South Asians in Toronto (Ontario), and found, in both cities, “noticeable differences in the age-standardized mortality rates depending on the proportion of the neighbourhood population who were Black in Montréal and South Asian in Toronto”. Black, South Asian, and Indigenous Canadians are more likely than white Canadians to have multiple illnesses such as diabetes, asthma, cancer or other conditions [35], and are therefore more at risk of a serious outcome from COVID-19. For instance, in terms of racial disparity, more cases of ischaemic stroke associated with COVID-19 were reported among African-Canadians than among other races [36].

Regarding First Nations communities on reserves (these statistics did not include First Nations individuals residing off reserve or in the territories), 51 cases were reported from March 8 to April 11, more than half in Québec [37]. Retrospectively, no deaths involving First Nations people were reported until October 2020 [38]. COVID-19 seemed to have lower impact among First Nations in Canada than among other Indigenous communities in other countries, and even among the general population [39].

### 2.4. Measures Taken and Evolution of the COVID-19′s First Wave

#### 2.4.1. At the Federal Level

On January 15, the Public Health Agency of Canada decided to implement and activate the Emergency Operation Centre to support the Canadian response to COVID-19 and triggered the Federal/Provincial/Territorial Public Health Response Plan for the Biological Events to provide elaborated coordination across the country.

From January 22, the Canadian government recommended to all travelers coming from Wuhan, China to self-isolate for 14 days [40]. A federal-provincial-territorial Special Advisory Committee on the Novel Coronavirus was created to advise Deputy Ministers of Health across Canada on the coordination, public health policy, and technical content related to the COVID-19 outbreak [41].

On March 5, the federal government created a Cabinet Committee in charge of ensuring whole-of-government leadership, coordination, and preparedness to limit the health, economic and social impacts of the COVID-19. Then, a series of public health measures aiming at slowing down community transmission of the COVID-19 were implemented country-wide: describing and encouraging to proper hand hygiene, respiratory etiquette and environmental cleaning in the home, and social distancing.

Some community-based measures were applied to reduce transmission within the community settings such as workplaces, schools, or public transportation (Figure 2); these measures included avoiding crowded spaces, school, or daycares, as well as increasing distancing between desks, closing schools, raising awareness about COVID-19, and evaluating whether these measures are respected in workplace. In addition, the government allocated CAD 1101 million to different sectors to sustain the Canadian response to the crisis [42]. Shortage of surgical masks made their use prioritized for healthcare workers, and not mandatory indoors for the general population before later on; yet the use of cloth masks was encouraged as a supplementary protection, especially when physical distancing was difficult [43].

After COVID-19 acquired its pandemic status (March 11), the Canadian government implemented several new measures; for instance, Canadian residents were advised to avoid all non-essential travel outside of Canada, travelers entering Canada had to self-isolate for 14 days even if they did not present any COVID-19 symptoms, with fines in case of non-respect. In the end of the study period, contact tracing was not performed massively, but different initiatives had started to develop tracing apps, and some provinces had organized their own contact tracing system by phone call as soon as late March in British Columbia [44]. On March 18, the authorities decided to implement a ban on foreign nationals from all countries, except the United States, from entering Canada. The Canada-U.S. border was closed to all non-essential travels [42].

#### 2.4.2. By Administrative Division

Each province or territory implements its own public health measures and decides to apply a public health emergency (which limits the federal government’s power to enforce health-related measures) or a state of emergency (which allows the provincial or territorial health officer to issue verbal orders without going through the normal legal procedure and with immediate effect) [45]. After COVID-19 was declared a pandemic, all provinces and territories declared at least a state of public health emergencies at different dates in late March, going on until various dates in April-May (Table 1); self-isolation measures, gathering restrictions, and fines in case of non-respect of the rules were also enforced at various degrees depending on the province. These measures evolved with time.

### 2.5. Expected or Observed Impact on the Country Economy

On March 18, the Government set up new economic measures to help industries and people face COVID-19. These measures were meant to provide up to CAD 27 billion to support Canadian workers and businesses (Canada’s COVID-19 Economic Response Plan). They also were meant to support people facing unemployment and centers or funds such as New Women’s Shelters, Sexual Assault Centers, or new Indigenous Community Support Fund [42]. From April 6, Canadian workers who got unemployed (or had their income reduced by at least 50%) due to COVID-19 received CAD 500 each week up to 16 weeks (as of April 2020) from the Canadian Emergency Benefits [42].

Due to social distancing, non-essential shop closure, and travel restriction, the spread of the coronavirus was forecast to particularly affect tourism (including “shopping-trip”, for which around 44 million people cross the USA-Canada border each year) and the oil sector. The closure of the Canada-US border to non-essential travels did not apply to food, goods, medical supplies and farm workers, still allowed to cross. Shops with online stores were expected to limit the impact of the virus on their activity [46].

### 2.6. Social Disruption and Indirect Public Health Consequences of COVID-19

As many public health officials around the world, the Canadian authorities implemented social distancing measures, considered to be one of the most reliable responses to avoid the spread of the COVID-19.

However, even in the short term, social distancing can be associated with psychological consequences such as overall distress, panic, emotional disturbances, and depression [47]. Even more, quarantine, as the most extreme social distancing measure, has been associated with stress, low mood, and insomnia. It has been observed that the longer the isolation period, the worse the mental health tended to be [47,48]. Long periods of lockdown could therefore lead to an increase of mental health disorders amongst the general population [47]. A survey by Statistic Canada in April–May 2020, just after our study period, reported that over half of the ca. 46,000 participants reported a poorer mental health since the onset of social distancing, and 88% had experienced at least one symptom of anxiety in the two weeks before participating to this survey [49]—a situation comparable to China’s [50]. Psychological distress can affect different domains of people’s lives, such as poorer work performance, family functioning, health services utilization, or social relations, and COVID-19 can be expected to trigger similar effects although this remains to be explored [48].

Beside mental health, the COVID-19 had other indirect consequences on public health and society, as soon as the first wave. For instance, an increased use of cannabis (a legal use in Canada) was associated with self-isolation [51]. After the population early linked COVID-19 to China and Asia, racism towards East Asian Canadians abruptly increased [52], as illustrated by increased hate crimes [53].

## 3. Discussion and Conclusions

To respond to the public health threat caused by the COVID-19, the Canadian government implemented relatively early an infrastructure in collaboration with provincial and territorial governments. Canada’s response was structured around plans and guidance at different levels: collaboration at all levels of government, evidence-informed decision-making, and flexibility with the actions taken according to the new information available. In addition, the Government of Canada created dedicated legislation and financial resources such as the Cabinet Committee on the federal response to the COVID-19.

Canada adopted different public health measures to try containing the spread of the coronavirus by putting in place measures such as hand hygiene, self-isolation, social distancing, and lockdown, and a testing strategy to identify cases and to find close contacts to the confirmed positive cases. Other measures regarding global travel advisory have also been added. Across Canada’s administrative divisions, different initial responses to the COVID-19 pandemic were enforced and different epidemiological dynamics were observed, with high death rates in the most densely populated regions. Research on the factors controlling the spread of COVID-19 in each Canadian administrative division is warrantied, and will allow for generating evidence-based recommendations suitable for this country both at the federal and provincial or territorial levels.

In the beginning of the first wave (January–April 2020), the available data showed slower hospitalization and death rates in Canada than in the USA or the European Union, both at national level and at the provincial or territorial level; that was even in the most populated provinces of Canada, despite the high mortality in the long-term care facilities observed in Québec and Ontario. Such inter-country comparisons may be important to understand how to control COVID-19 [54], and possible future epidemics of infectious diseases as they are thought to increase in the near future [55].

This case study focuses on the very beginning of the first wave; it was limited by the available peer-reviewed published scientific literature and contains facts or numbers from a gray literature that other non-governmental associations have compiled for Canada [56] and that may not be available anymore in the future. For this reason, we hope this case study will provide a useful archive and useful links to other valuable resources on the COVID-19 management in Canada during the first wave.

## Figures and Tables

**Table 1:** Type and date of emergency state declared by the Canadian provinces and territories.

Provinces/Territories	Emergency State	Precisions
**Alberta**	State of public health emergency March 17	Gatherings of no more than 50 first and then only 15; closure of public area and popular facilities (gym, museums, arenas, galleries), self-isolation for 14 days after an international travel and 10 days for people with symptoms.
**British Columbia**	State of emergency March 18—This state of emergency will be extended or rescinded as necessary.	Gatherings of no more than 50; financial penalties for business and individuals; financial penalties for businesses and individuals violating public health directives (Vancouver)
**Manitoba**	State of emergency March 20—30 days and could be extended if necessary.	Gatherings to maximum 10
**New Brunswick**	State of emergency March 19—until April 15 and could be extended if necessary.	Gatherings of no more than 10; closure of all retail operations, except for essential services (Public inquiries, Veterinary Field and Laboratory Services, Motor Vehicle Safety Enforcement…); financial penalties
**Newfoundland and Labrador**	Public health emergency March 18—until April 15 and could be extended if necessary.	Gatherings of no more than 10; no-essential businesses closure; fine or jail time
**Nova Scotia**	State of emergency March 22—until April 19 and could be extended if necessary.	Gatherings of no more than 5; Some non-essential services remain open with social distancing
**Ontario**	State of emergency March 17- until April 14 and extended for 28 days.	Gatherings of no more than 5; closure of public facilities and non-essential businesses
**Prince Edward Island**	State of public health emergency March 16—30 days and could be extended if necessary.	Encourage islanders to avoid social gatherings; fines up to CAD10,000
**Quebec**	Public health emergency, March 13—until May 4.	Social gatherings are prohibited; fines
**Saskatchewan**	State of emergency March 18—until April 15 and could be extended if necessary.	Gatherings of no more than 10; closure of non-essential shops; individual fines
**Northwest Territories**	State of public health emergency March 18—until April 15 and could be extended if necessary.	Gatherings of no more than 10
**Nunavut**	State of public health emergency March 18—until April 16 and could be extended if necessary.	Public gatherings prohibited
**Yukon**	State of emergency March 27—until April 30 and could be extended if necessary.	Gatherings of no more than 10; Reduce restaurant capacity to 50%
